# Digital Nudges Among New Gym Members: Controlled Formative Evaluation of Gym Attendance, Exercise Self-Efficacy, Habit Strength, and Exercise Adherence

**DOI:** 10.2196/93800

**Published:** 2026-10-16

**Authors:** Maor Gabay, Ofer Levi, Lupsa Marius Matichescu, Mihaela Oravitan

**Affiliations:** 1 Faculty of Physical Education and Sports, West University of Timişoara, B-dul Vasile Pârvan nr. 4, Timișoara, Timiș County, 300223, Romania, 0040 256 592129; 2 Department of Physical Education and Sports, Kaye Academic College of Education, Beer Sheva, Southern District, Israel; 3 Open University of Israel, Raanana, Central District, Israel; 4 Faculty of Sociology and Social Work, West University of Timişoara, Timisoara, Timiș County, Romania

**Keywords:** digital health, digital nudging, physical activity promotion, exercise adherence, behavior change intervention, habit formation, mHealth, mobile health

## Abstract

**Background:**

Digital nudging strategies are increasingly implemented to promote physical activity; yet, evidence from real-world fitness settings remains limited. The early membership period in fitness centers is characterized by high dropout rates, emphasizing the need for formative evaluations that clarify behavioral mechanisms underlying exercise engagement.

**Objective:**

This study aimed to evaluate the formative impact of WhatsApp (Meta Platforms, Inc)-based digital nudges on objectively measured weekly gym visits among new fitness club members and to examine behavioral determinants associated with exercise engagement.

**Methods:**

This controlled intervention study used a 2-parallel-group design with quasi-random individual allocation conducted separately within 2 fitness clubs. The complete-case sample comprised 61 new gym members (mean age 29.05, SD 8.06 years; 21/61, 34.4% female), including 27 participants in the intervention group and 34 in the control group. During the 3-week intervention period, participants in the intervention group received brief digital nudges through designated WhatsApp groups. The messages were developed according to the Easy, Attractive, Social, and Timely (EAST) framework. Participants in the control group received no nudge messages. Gym visits were recorded electronically over 12 weeks through the fitness clubs’ computerized entry systems. Self-reported measures included exercise self-efficacy, habit strength, and exercise adherence. Statistical analyses included descriptive statistics, longitudinal analysis of weekly gym attendance using generalized estimating equations (GEE), correlation analyses, and exploratory regression analysis.

**Results:**

Longitudinal GEE analysis showed a significant decline in weekly gym attendance over time (b=–0.074, SE 0.016; *P*<.001, incidence rate ratio [IRR]=0.929), corresponding to an approximately 7.1% decrease in expected weekly visits per study week. However, the group×week interaction was not significant (b=.009, SE 0.022; *P*=.68, IRR=1.009). Participants attending the gym at least twice weekly reported greater exercise habit strength (mean 33.89, SD 6.49 vs mean 28.70*,* SD 9.05; *P*=.02) and greater baseline exercise self-efficacy (mean 71.37, SD 12.50 vs mean 57.24, SD 14.61; *P*=.001). Mean weekly gym visits were positively correlated with exercise habit strength (*r*=0.444), exercise self-efficacy (*r*=0.498), and exercise adherence (*r*=0.507; all *P*<.001). In the exploratory regression analysis, exercise adherence was the only variable retained, and the model explained 25.7% of the variance in mean weekly gym visits (*R*^2^=.257; *P*<.001).

**Conclusions:**

Weekly gym attendance declined significantly over the 12-week follow-up period, but the rate of decline did not differ significantly between the intervention and control groups. However, exercise adherence, exercise self-efficacy, and exercise habit strength were positively associated with more frequent attendance, and participants attending at least twice weekly reported greater self-efficacy and stronger exercise habits. These exploratory findings identify potentially relevant behavioral factors for future adherence interventions.

## Introduction

### Background

Among the major challenges facing the fitness industry are high membership turnover, low member loyalty, and difficulties in maintaining long-term retention. In the Spanish fitness industry, an estimated 19% to 24% of consumers discontinue participation in sport after leaving their fitness center [1]. Although this estimate is specific to Spain, member dropout represents a broader concern because fitness centers provide an important setting for regular physical activity, with implications for both the fitness sector and public health [2]. Maintaining adherence to long-term exercise is a challenging task. Approximately 50% of people reduce such activity after the first month [3], and 40%‐65% of individuals who begin an exercise program are predicted to quit during the first 3 to 6 months [4]. In a study that included 5240 individuals (3084 women and 2164 men), Sperandei et al [5] reported that half of new gym members quit before 3 months of active participation. Moreover, Gjestvang et al [3] reported that only 37% of participants reported regular adherence during their first year of fitness club membership.

People drop out of sport programs for a variety of reasons, including high membership fees, the perception that participation requires a substantial time commitment, engagement in other hobbies, relocation, family responsibilities, work commitments, and health problems. Moreover, in a recent comprehensive narrative review [6], our research team identified several factors associated with exercise adherence within fitness facilities. These factors included social and professional support, habit formation and previous exercise behavior, motivation and perceived barriers, and personal characteristics such as age and gender. Social and professional support may facilitate continued participation by providing encouragement, guidance, and accountability, whereas habit formation and previous exercise behavior may contribute to greater consistency by helping individuals integrate exercise into their regular routines. Motivation and perceived barriers may further influence whether individuals are willing and able to maintain regular participation, while personal characteristics may shape exercise goals, preferences, and the practical challenges associated with sustained adherence.

Our review also introduced practical implementation strategies designed to improve consistency and adherence. These strategies included recommendations regarding optimal weekly training frequency, guidance for developing physical activity habits, particularly among beginners, the promotion of a supportive social and professional environment, and emphasis on the role of fitness professionals in addressing trainees’ psychological needs and preventing dropout among vulnerable populations. These strategies informed the development of the digital nudges used in this study. Specifically, the messages encouraged regular weekly attendance, supported the development of consistent exercise routines, highlighted the value of social and professional support, and emphasized the role of fitness professionals in helping new members overcome barriers to continued participation.

In addition, in a recent study involving 460 participants [7], we observed that exercise adherence among individuals who participated in resistance training was greater than among those who participated in flexibility or aerobic training. The study also showed that women reported body-toning and weight-loss goals more frequently than men, that individuals aged 45 years and older were more health-oriented than younger individuals, and that those who had maintained an exercise routine for more than 12 months reported greater self-efficacy than those who had exercised for a shorter period. In accordance with the findings of our previous research and other studies, this study aimed to better understand exercise adherence among new gym members by implementing an intervention and follow-up program at 2 fitness clubs. Specifically, we investigated the effect of digital nudges, delivered as WhatsApp (Meta Platforms, Inc) messages, on the weekly frequency of gym visits. We also examined the associations of self-reported exercise self-efficacy, exercise habit strength, and exercise adherence with weekly gym attendance among new gym members.

### Overview

Nudges are tools that are rooted in psychology; they can be strategically crafted to facilitate behavioral changes, with the aim of improving health and well-being. They can influence decision-making by providing information, dispelling misconceptions about social norms, modifying the presentation of different choices, and implementing default options. The underlying principle in these instances is to make the “better” option appear more visible or convenient, as that option is deemed superior for maximizing future health, wealth, and overall well-being [8].

In a 2019 scoping review, Forberger et al [9] examined the use of nudging interventions to promote physical activity in the general population. The findings suggested that nudging may represent a promising approach for increasing physical activity by making the desired behavior more salient, reducing the cognitive effort required to act, and providing prompts at relevant decision-making moments. Nevertheless, the review also identified substantial research gaps and highlighted several opportunities that remain insufficiently explored. These findings indicate a need for further investigation and more comprehensive evaluation of the potential applications of nudging to promote physical activity across diverse populations. In addition, a systematic review examining the effectiveness of nudges for improving self-management among patients with chronic diseases found that reminders, planning prompts, small financial incentives, and feedback were among the most frequently evaluated strategies. Overall, these interventions generally had positive effects on self-management outcomes, including exercise and medication adherence [10].

Time constraints and procrastination have been identified as potential barriers to physical activity participation [11]. To address these barriers, push-notification reminders may provide a practical strategy for encouraging physical activity by prompting timely and actionable responses. This approach uses mobile technology to support behavior at relevant moments and may help individuals translate their intentions into action. Accordingly, Bond et al [12] demonstrated the effectiveness of a smartphone-based intervention that incorporated instructions for activity to significantly reduce prolonged sitting time among individuals classified as overweight or obese. Furthermore, Andersen et al [13] reported that the introduction of clear email-based messages aimed at encouraging vigorous physical activity served as an effective strategy for enhancing aerobic fitness among office workers. Notably, the weekly volume of stair climbing reported in that study corresponded to approximately half of the weekly vigorous-intensity physical activity recommended by the American College of Sports Medicine and the American Heart Association. Evidence from gym-based studies has also supported the potential effectiveness of nudging interventions, including email reminders, in promoting attendance [14,15]. These reminders appeared to be particularly effective among individuals with initially low attendance, contributing to an approximately 27% increase in gym participation [14]. Taken together, these findings highlight the potential relevance of behavioral economics–based interventions, particularly nudges, for promoting physical activity and informing health promotion initiatives.

Nevertheless, evidence regarding the effectiveness of digital nudges among newly enrolled fitness club members remains limited. In particular, relatively little is known about whether a brief WhatsApp-based intervention can support objectively recorded gym attendance during the early stages of membership or how exercise self-efficacy, exercise habit strength, and exercise adherence are associated with attendance during this period. Accordingly, this study investigated whether a digital nudging intervention was associated with differences in the frequency of gym attendance. We also examined the associations between self-reported exercise habit strength, exercise self-efficacy, exercise adherence, and the maintenance of a consistent gym routine.

Nudge theory is well established and has been applied to encourage physical activity [9,11-13]. Previous evidence has also demonstrated the potential effectiveness of similar interventions among fitness club members [14,15]. In addition, Semrl and Matei [16] reported that a substantial proportion of fitness club dropout occurs between the fourth and eighth weeks of membership. Based on this evidence, we developed a digital nudging intervention guided by the principles of an evidence-based behavioral framework [17]. Consequently, we formulated the hypotheses mentioned below.

### Hypotheses

#### Hypothesis 1

Compared with those in the control group, new gym members participating in the intervention program will exhibit a higher frequency of weekly gym visits.

Recent meta-analyses using the Self-Report Habit Index scale have explored the associations between habit strength and health behaviors, emphasizing the critical role of habits in shaping health-related actions [18]. Evidence suggests that individuals who engage in exercise at least twice per week are more likely to develop consistent exercise habits, as training frequencies of 2‐4 sessions per week have been identified as essential for habit formation [6]. Conversely, an insufficient training frequency may substantially increase the risk of gym dropout [19]. Thus, we believe hypothesis 2.

#### Hypothesis 2

Individuals who attend the gym more than twice per week, on average, will report higher levels of exercise habit strength than those who exercise less frequently.

In addition, perceived self-efficacy reflects an individual’s confidence in their capacity to successfully execute the actions required to manage unfamiliar future scenarios [20]. Similarly, self-efficacy and beliefs related to physical activity are critical determinants in motivating individuals to engage in and sustain regular physical activity [21-23]. Accordingly, we believe the following:

#### Hypothesis 3

Individuals who exercise more than twice per week demonstrate higher levels of exercise self-efficacy than those who exercise less frequently.

Adherence is a critical component of behavioral change [24]. Hence, hypothesis 4 is listed below.

#### Hypothesis 4

Exercise adherence will serve as a significant predictor of weekly gym visits.

## Methods

### Study Design and Setting

This controlled intervention study used a 2-parallel-group design with quasi-random individual allocation conducted separately within each of the 2 participating fitness clubs. Participants were allocated to either an intervention group or a control group. The intervention group received text- and graphic-based nudges through designated WhatsApp groups to encourage regular gym attendance and adherence to exercise. The control group received no nudge messages but completed the same study assessments as the intervention group.

The study assessments comprised electronically recorded gym visits and self-report questionnaires administered at 4 time points: T1, on the first day of the study; T2, 3 weeks after the start of the study, at the beginning of week 4; T3, 6 weeks after the start of the study, at the beginning of week 7; and T4, 12 weeks after the start of the study (Figure 1).

**Figure 1.**
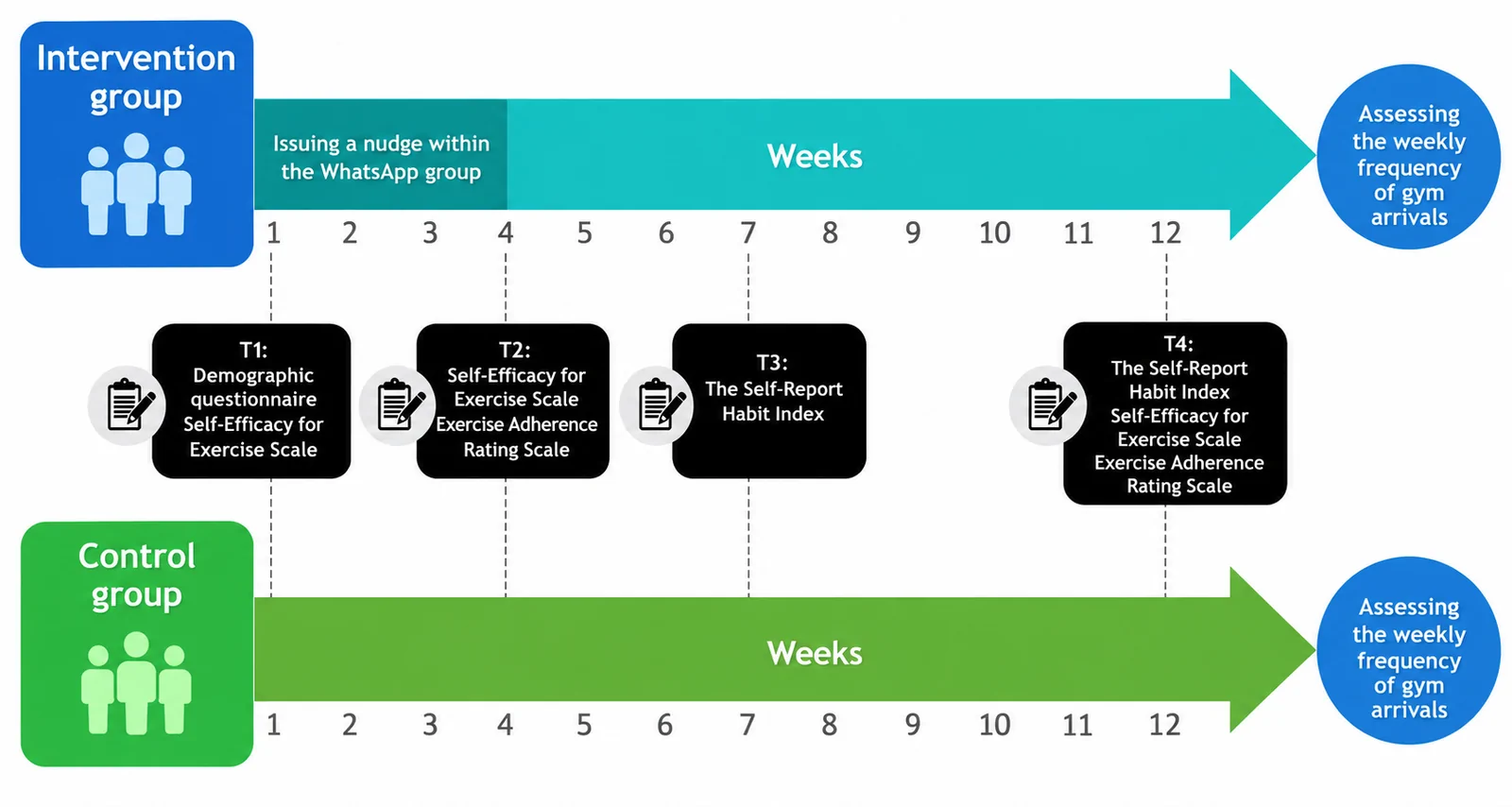
Study timeline and assessment schedule. T1: baseline; T2: week 3; T3: week 6; T4: week 12.

Participants in the intervention condition received WhatsApp-based text and graphic nudges during weeks 1-3, whereas participants in the control condition received no nudge messages. Electronically recorded gym visits were assessed weekly for 12 weeks. Self-report questionnaires were administered at T1, T2, T3, and T4.

During the 3-week intervention period, nudge messages containing text and graphics were delivered exclusively to the intervention groups through WhatsApp. Questionnaires were administered online to participants in both study conditions at the prespecified time points. Participants’ national identification numbers were used as unique identifiers to link questionnaire responses across the 4 assessment points and to the computerized gym-entry records.

The study was conducted at 2 fitness clubs in southern Israel, located in Ofakim and Be’er Sheva. Data were collected between January 17 and April 21, 2024. The first site, Space Ofakim (gym 1), was a branch of the Space fitness-club chain in Ofakim. The second site was the fitness facility of the Ben-Gurion University Sports Center (gym 2) in Be’er Sheva.

### Participants and Recruitment

Each fitness club provided the principal investigator (MG) with a list of new members who had joined during the 3 weeks preceding the beginning of the study. Potential participants were contacted by telephone by the principal investigator and invited to participate. The study procedures, voluntary nature of participation, right to decline participation without disadvantage, and right to withdraw at any time were explained during the telephone call and reiterated in a written message at the beginning of the study.

Individuals who verbally agreed to participate were added to the WhatsApp group corresponding to their previously assigned study condition. They then received the electronic informed consent form and the T1 baseline questionnaire. Formal informed consent was documented electronically through a mandatory consent item included before the T1 questionnaire. No intervention messages were delivered before electronic informed consent was obtained.

New fitness-club members who had enrolled no more than 3 weeks before the beginning of the study were recruited. This eligibility period was selected on the basis of findings reported by Semrl and Matei [16], who identified a substantial decline in attendance among new gym members after the fourth week of membership. Recruitment and baseline assessment were therefore conducted before participants reached their fourth week of membership.

The inclusion criteria were (1) age 18 years or older; (2) general good health, documented by a health declaration or, when required, a medical certificate submitted to the fitness club; (3) ability to speak and read Hebrew; and (4) ability to read, understand, and complete the study questionnaires and comprehend the WhatsApp messages used in the intervention.

Individuals who did not meet these criteria were not eligible to participate. Participants who did not complete all 4 questionnaires were excluded from the complete-case questionnaire analyses. The numbers of participants available at each assessment point and included in each analysis are presented in Figures 1 and 2, and Table 1.

The principal investigator administered the questionnaires and intervention messages, managed data collection, and was aware of participants’ group assignments. The statistical analyses were conducted by members of the research team who were also aware of the study group assignments.

**Figure 2.**
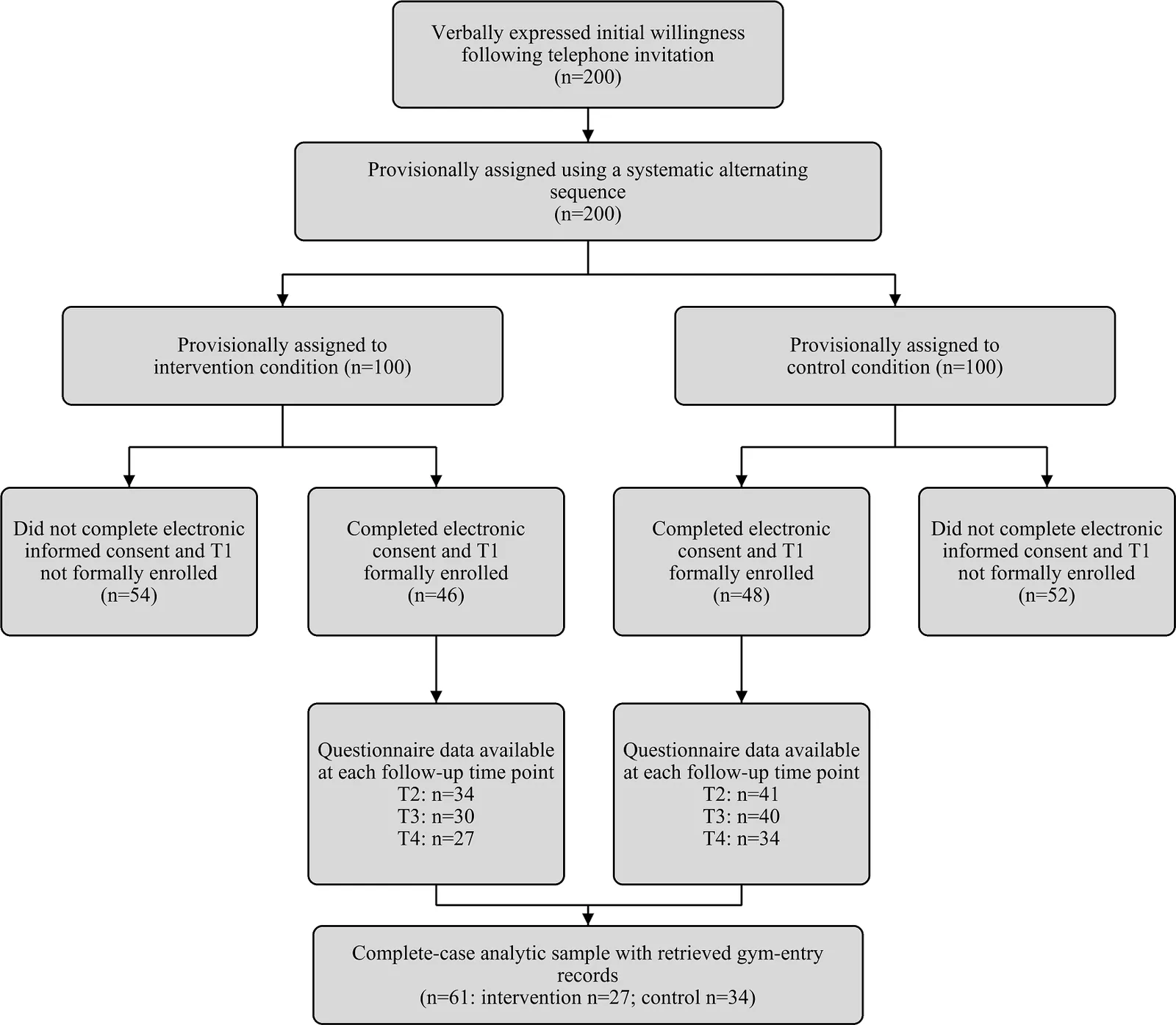
Participant flow through provisional assignment, formal enrollment, follow-up, and complete-case analysis. T1 baseline; T2, week 3; T3, week 6; T4, week 12.

**Table 1.** Participants providing questionnaire data at each assessment by fitness club site and study group^a^.

Fitness club and study group	T1^b^, n	T2^c^, n	T3^d^, n	T4^e^, n	Complete-case retention, n/N (%)
Gym 1					
Control group	23	23	23	18	18/23 (78.3)
Intervention group	22	15	12	9	9/22 (40.9)
Gym 2					
Control group	25	18	17	16	16/25 (64)
Intervention group	24	19	18	18	18/24 (75)
Control total	48	41	40	34	34/48 (70.8)
Intervention total	46	34	30	27	27/46 (58.7)
Overall	94	75	70	61	61/94 (64.9)

a Values represent the number of participants providing questionnaire data at each assessment. Complete-case retention was calculated relative to the number of participants formally enrolled at T1.

b T1: baseline.

c T2: week 3.

d T3: week 6.

e T4: week 12.

### Allocation and Group Assignment

Before recruitment began, the principal investigator received from each fitness club a list extracted from its computerized customer relationship management system containing all new members who had joined during the preceding 3 weeks. Allocation was performed separately within each fitness club. Participants on each site-specific list were provisionally assigned to the intervention or control condition according to a systematic alternating sequence. The principal investigator established and implemented the allocation sequence and determined the first assignment at each site. Thereafter, participants were assigned alternately to the intervention and control conditions in the order in which they appeared on the site-specific list. Because the allocation sequence was predetermined and systematic, subsequent assignments were predictable and allocation concealment was not possible. Accordingly, the allocation procedure was described as quasi-random rather than randomized.

### Research Nudges

Within the intervention group’s WhatsApp platform, nudge messages featuring graphics and informative content were disseminated, drawing on insights from our previous studies [6,7]. The objective was to foster adherence among new members of the fitness club. This approach is grounded in the sustained impact that has been observed at 3 months posttherapy for low-cost interventions such as text messages and reminders. These interventions have the potential to increase weekly physical activity frequencies across a diverse demographic spectrum that includes women, men, both new and returning members, and students and nonstudents [25].

Calzolari and Nardotto [26] demonstrated the significant impact of straightforward weekly reminders on increasing gym users’ physical activity levels over an extended period, with evidence supporting the formation of habits induced by reminders. Despite existing insights into the effectiveness of text message reminders, to our knowledge, no controlled intervention studies were identified that used a framework similar to this study, which uses messages providing practical applications to enhance adherence and consistency among new fitness club members. Notably, the use of group dynamics through the WhatsApp app for such messaging interventions represents a unique and unexplored approach in the literature.

### Use of WhatsApp and Delivery of the Digital Nudges

In contrast to previous studies that used email reminders [14,15], this study delivered digital nudges through designated WhatsApp groups. WhatsApp was selected because it has become one of the most widely used instant-messaging apps among mobile device users worldwide and is particularly popular in Israel. According to a survey conducted by QualiTest in 2018, 97% of respondents in Israel reported regularly using WhatsApp on their smartphones, compared with 78% for Facebook (Meta Platforms, Inc.), 34% for Instagram (Meta Platforms, Inc.), and 7% for Twitter (subsequently rebranded as X; X Corp) [27].

WhatsApp groups offered several practical advantages for the implementation of the study, including the ability to manage participants, enable voluntary withdrawal, distribute messages simultaneously to multiple participants, and provide links to the online questionnaires. The platform also supported the delivery of content in several formats, including text and images. In this study, the digital nudges consisted primarily of concise written messages accompanied by visual elements, including images, vector graphics, and emojis. Figure 3 presents an example of a nudge addressing social and professional support.

**Figure 3.**
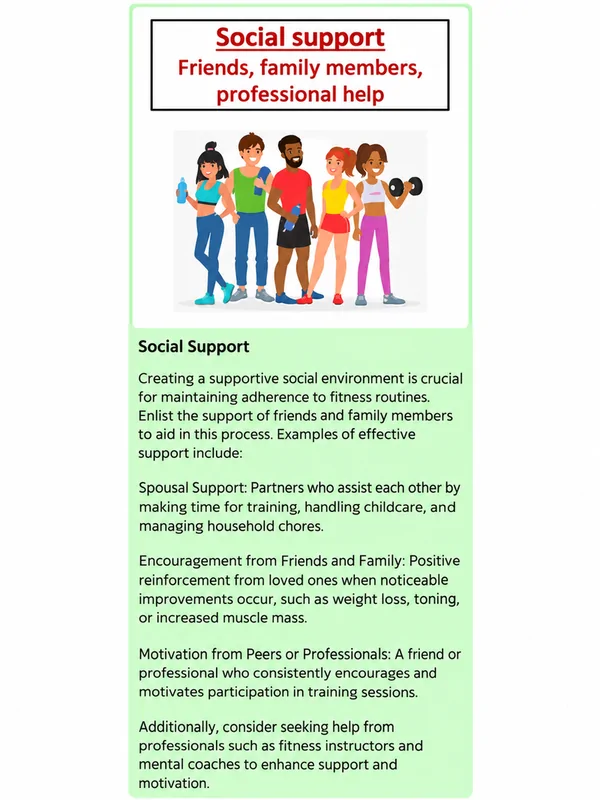
Example of a WhatsApp-based digital nudge illustrating the “Make It Social” principle of the Easy, Attractive, Social, and Timely (EAST) framework.

The message presented social support from friends, family members, training partners, instructors, and coaches as a potential strategy for supporting regular gym attendance and continued exercise participation. This example illustrates how the “Make It Social” principle of the Easy, Attractive, Social, and Timely (EAST) framework was reflected in the intervention content.

The nudges were delivered manually by the principal investigator through the designated intervention-group WhatsApp groups during the 3-week intervention period. Messages were sent at intervals of 1 to 2 days, primarily during weekday afternoons and on Friday mornings. Participants in the control condition received no nudge messages, although both groups received links to the study questionnaires at the prespecified assessment time points. The theoretical basis of the intervention and the application of the EAST principles to the content, presentation, social context, and timing of the messages are described in the following section.

### EAST Framework

In addition, we used the EAST framework, an evidence-based approach, to effectively facilitate behavioral change. Developed by the Behavioral Insights Team, this model aims to assist practitioners and policymakers in the practical application of behavioral insights [17]. The framework centers on 4 key principles (Figure 4) that aim at facilitating behavior change: EAST.

**Figure 4.**
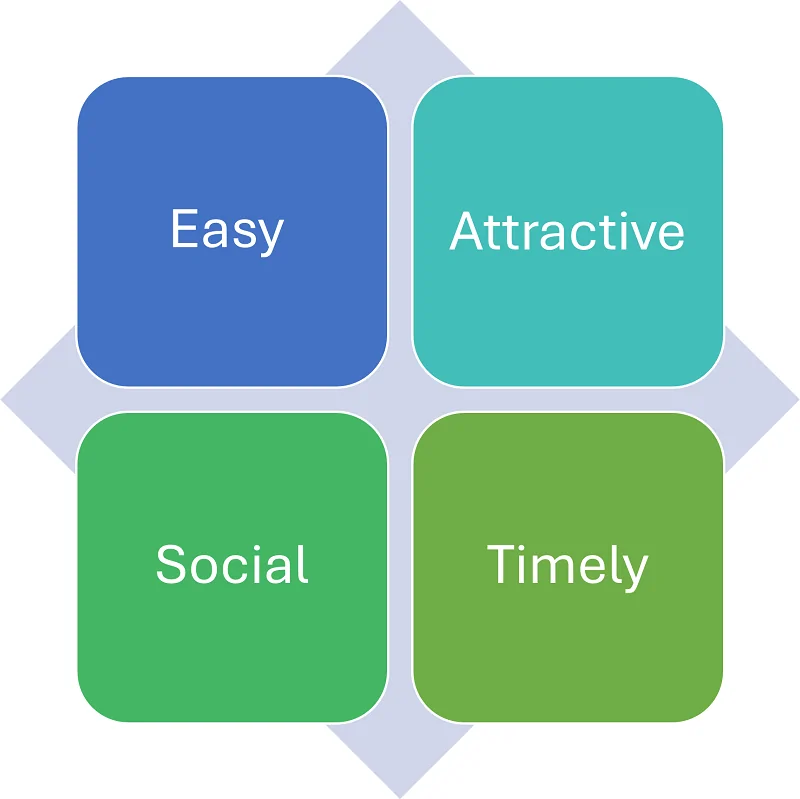
The Easy, Attractive, Social, and Timely (EAST) framework used to guide the development of the WhatsApp-based digital nudges delivered during the intervention [18].

The first principle, “make it easy,” emphasizes the need for simplicity in the process. Given the inclination to opt for the default option, designating the preferred behavior as the default can enhance its adoption. Alternatively, minimizing the effort needed to adopt a desired behavior is also effective. Clear communication is crucial, as a straightforward message often leads to a significant increase in the response rate. If the desired behavior change is intricate, it should be deconstructed into simpler, more manageable actions.

The second principle, “make it attractive,” involves drawing attention to communication. Behavior change is more likely to occur when attention is directed toward the desired action. Visual techniques, such as the use of color and pictures, can attract attention. Additionally, using rewards and sanctions tends to amplify effects, with rewards supporting the desired behavior change and sanctions deterring taking no action.

“Make it social,” the third principle, taps into collective action. By illustrating how others engage in the desired behavior, individuals can be encouraged to follow suit, especially if the others are part of the person’s network.

The final principle, “make it timely,” considers when individuals are most likely to be receptive, along with the timing of costs and benefits. Leveraging disruptions in habitual routines can be effective, and immediate impacts tend to have greater effects than delayed impacts do [17].

### Application of the EAST Framework in Our Study

#### Overview

The digital nudges were designed to incorporate one or more principles of the EAST framework, depending on the behavioral objective of each message.

#### Make It Easy

To reduce the effort required to receive and understand the intervention content, participants were contacted through WhatsApp, an app that was already installed on their smartphones. After providing verbal agreement to participate, they were added to the WhatsApp group corresponding to their allocated condition without being required to complete an additional joining procedure. The intervention messages were concise, clearly structured, and written in accessible language. In addition, the nudges presented practical and manageable recommendations intended to simplify exercise-related decision-making and support regular gym attendance [11,17].

#### Make it Attractive

The messages were designed to draw attention to the recommended behavior through visually appealing and audience-appropriate content [11]. Consistent with the EAST framework [17], the messages used concise headings, bullet points, boxed content, visual emphasis, emojis, and images containing vector graphics. These elements were intended to increase the salience of the messages and facilitate rapid comprehension of their central recommendations.

#### Make it social

The social principle was incorporated primarily through messages emphasizing the potential contribution of social and professional support to continued exercise participation. Participants were encouraged to seek encouragement and practical support from friends, family members, training partners, fitness instructors, and coaches. The use of designated WhatsApp groups also created a shared communication environment in which participants could observe that other new gym members were taking part in the same study. An example of a nudge addressing social and professional support is presented in Figure 3.

#### Make it Timely

The timing of the messages was determined on the basis of the average gym-entry times recorded for the target population, because the potential effectiveness of nudging may depend on delivering prompts at relevant moments [11]. Nudges were therefore sent primarily during weekday afternoons and on Friday mornings. Friday-morning delivery reflected the earlier closing times of fitness clubs in Israel on Fridays. To maintain regular exposure while limiting excessive messaging, the nudges were delivered at intervals of 1 to 2 days during the 3-week intervention period.

### Instruments and Measures

#### Exercise Self-Efficacy

The self-efficacy for exercise (SEE) scale [28] measures self-efficacy in physical exercise and consists of 9 items (α=0.92). To examine the average self-efficacy reports throughout the study period, SEE was measured at 3 different time points (T1, T2, and T4).

#### Habit Strength

The self-reported habit index is a self-reported measure for assessing habit strength [29]. This questionnaire is structured around a core question (stem) and varying responses tailored by the researcher to suit specific study requirements. For example, in this study, the stem question was “Physical exercise is something that...,” followed by responses such as “I do automatically,” “I do frequently,” and “I do without consciously remembering,” among others. The participants rated their level of agreement via a 5-point Likert scale for each item. An average score across the items was subsequently computed to gauge the strength of the habit (α=0.92). This measurement tool was measured at time points T3 and T4. The decision to test the strength of the habit only after 6 weeks (T3) stems from the findings of Kaushal and Rhodes [30], who reported that it takes at least 6 weeks to develop an exercise habit; thus, we wanted to allow for this period of time. Additionally, habit strength was measured again after 12 weeks (T4) to obtain the average habit strength of the subjects.

#### Exercise Adherence

We used the Exercise Adherence Rating Scale (EARS) to assess exercise adherence and factors that may help or hinder it [31]. The EARS was originally developed to assess adherence to prescribed home exercise and consists of 3 sections. Section A records details of the prescribed exercise program and was not administered in this study. Sections B and C contain 6 adherence-behavior items and 10 help-or-hindrance items, respectively. Although Section B was validated as a 6-item adherence measure, the original development study also explicitly described a scoring procedure in which all 16 items were summed. Accordingly, responses were rated on a 5-point Likert scale, positively phrased items were reverse-scored, and all 16 items were summed, with higher scores indicating better adherence. We refer to this measure as the 16-item EARS composite. It demonstrated good internal consistency at T2 (Cronbach α=0.81) and acceptable internal consistency at T4 (α=0.77).

#### Frequency of Gym Visits

The fitness clubs’ automatic entrance control determines the frequency of arrivals. The frequency of arrival at the gym was verified by a chip given to each participant, which was passed through at the gym entry point to access the gym.

#### Statistical Analyses

Statistical analyses were conducted using International Business Machines (IBM SPSS) Statistics version 23.0 (IBM Corp), and MATLAB R2026a (Version 26.1; The MathWorks, Inc). Descriptive statistics were used to summarize participant characteristics and study variables. Weekly gym attendance was analyzed longitudinally using generalized estimating equations (GEE), with the weekly number of gym visits specified as a count outcome using a Poisson distribution with a log link. The model included study group (intervention vs control), study week (Weeks 1‐12), fitness-club site (gym 1 vs gym 2), and the group×week interaction. Repeated weekly observations were clustered within participants, and an exchangeable working correlation structure with robust standard errors was specified. The 12 weeks were treated as relative study time points at both fitness-club sites, irrespective of the calendar dates on which data collection began. Model coefficients are reported with robust SEs, *P* values, and incidence rate ratios (IRRs).

Pearson correlation analyses were used to examine associations among average weekly gym visits, exercise habit strength, exercise self-efficacy, and exercise adherence. An exploratory stepwise multiple linear regression analysis was conducted to examine behavioral and demographic variables associated with average weekly gym visits. Statistical significance was set at *P*<.05, and 95% CIs were reported for the principal estimates.

#### Sample Size

An a priori power analysis was conducted using G*Power (version 3.1.9.7: Heinrich Heine University Düsseldorf) [32], with α=.05 and 90% power. The original power analysis was based on the statistical analyses specified in the initial study plan and indicated required sample sizes of 172 participants for the primary between-group comparison, 112 for the correlation analyses, and 88 for a multiple regression model. Accordingly, the recruitment target was set at 200 participants. The final analytic sample included 61 participants and therefore did not achieve the originally planned statistical power. No separate a priori power calculation was performed for the longitudinal GEE analysis introduced in the revised manuscript.

### Ethical Considerations

The research protocol, including the study questionnaires, was approved by the Ethics Committee of the West University of Timișoara (approval number 15227/7.03.2023). All the participants signed a consent form before participating in the study. Instruments that were not previously available in Hebrew were translated from English into Hebrew using a forward- and back-translation procedure [33,34].

## Results

### Participant Flow and Sample Characteristics

Following telephone recruitment, 200 individuals verbally expressed initial willingness to participate and were provisionally assigned to a study condition. Of these, 94 completed the electronic informed-consent form and the T1 questionnaire and were formally enrolled: 46 in the intervention group and 48 in the control group. The remaining 106 individuals did not complete formal enrollment. Questionnaire data were available for 75 participants at T2 (intervention, n=34; control, n=41), 70 at T3 (intervention, n=30; control, n=40), and 61 at T4 (intervention, n=27; control, n=34). These 61 participants completed all 4 assessments and were included in the complete-case analyses. The complete-case retention rate among formally enrolled participants was 64.9% (61/94) overall, 58.7% (27/46) in the intervention group, and 70.8% (34/48) in the control group. Participant flow through provisional assignment, formal enrollment, follow-up, and complete-case analysis is presented in Figure 2. In Figure 2 the 200 individuals verbally expressed initial willingness during telephone recruitment and were provisionally assigned before formal enrollment. Formal enrollment required electronic informed consent and completion of the T1 questionnaire (n=94); no intervention messages were delivered before consent. Specific reasons for subsequent questionnaire noncompletion were not systematically recorded. Assessment-specific participant counts by fitness-club site and study group are presented in Table 1.
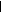


The following demographic characteristics refer to the 61 participants included in the complete-case analytic sample (intervention, n=27; control, n=34), rather than to all 94 formally enrolled participants. Their mean age was 29.05 (SD 8.06) years, and 21 participants (21/60, 34.4%) were female. The highest level of education was high school for 31 participants (31/61, 50.8%) and higher education for 30 participants (30/61, 49.2%). Regarding employment status, 26 participants (26/61, 42.6%) reported full-time employment, (18/61, 29.5%) part-time employment, and (17/61, 27.9%) were not currently employed. A total of 40 participants (40/61**,** 65.6%) were single, (20/61, 32.8%) were married, and (1/61, 1.6%) was divorced or separated. Additional characteristics of the complete-case sample by study group are presented in Table 2.

**Table 2.** Characteristics of study participants by intervention and control group^a^.

Characteristics	Control (n=34)	Intervention (n=27)
Age, mean (SD)	27.5 (5.9)	31 (10)
Sex, n (%)		
Male	21 (61.8)	19 (70.4)
Female	13 (38.2)	8 (29.6)
Education, n (%)		
High school education	17 (50)	14 (51.9)
Academic education	17 (50)	13 (48.1)
Employment, n (%)		
Employed	24 (70.6)	24 (88.9)
Self-employed	10 (29.4)	3 (11.1)
Scope of employment, n (%)		
Not working at the moment	9 (26.5)	8 (29.6)
Part-time	9 (26.5)	9 (33.3)
Full-time	16 (47.1)	10 (37.0)
Marital status, n (%)		
Single	23 (67.6)	17 (63.0)
Married	10 (29.4)	10 (37.0)
Divorced or separated	1 (2.9)	0 (0)
Body weight (kg), mean (SD)	79.5 (17.2)	76.5 (14.4)
Height (cm), mean (SD)	174.6 (8.9)	173.8 (8)

a Values are presented as mean (SD) for continuous variables and n (%) for categorical variables. Control group, n=34; intervention group, n=27. Percentages were calculated within each study group.

The control and intervention groups were relatively homogeneous with respect to participant characteristics. Both groups had a balanced distribution of educational backgrounds, with approximately half of each group having a high school education (control: 17/34, 50.0%; intervention: 14/27, 51.9%) and the other half having higher academic education (control: 17/34, 50.0%; intervention: 13/27, 48.1%). Additionally, both groups presented similar proportions of individuals not currently employed (control: 9/34, 26.5%; intervention: 8/27, 29.6%), working part-time (control: 9/34, 26.5%; intervention: 9/27, 33.3%), and working full-time (control: 16/34, 47.1%; intervention: 10/27, 37.0%). The marital status distribution was also similar, with the majority being single (control: 23/34, 67.6%; intervention: 17/27, 63.0%) and a smaller percentage being married (control: 10/34, 29.4%; intervention: 10/27, 37.0%). These comparable demographic characteristics, along with similar mean body weights (control: 79.5, SD 17.2 kg; intervention: 76.5, SD 14.4 kg) and heights (control: 174.6, SD 8.9 cm; intervention: 173.8, SD 8 cm), confirmed that the differences between the groups were not significantly influenced by these basic participant characteristics.

### Longitudinal Analysis of Weekly Gym Attendance

A Poisson GEE model was used to examine weekly gym attendance across the 12-week follow-up period (Figure 5). The model included intervention group, study week, fitness-club site, and the group×week interaction. There was a significant decline in gym attendance over time (b=−0.074, SE 0.016; *P*<.001, IRR=0.929), indicating an approximately 7.1% reduction in the expected number of weekly gym visits for each additional study week. The group×week interaction was not significant (b=0.009, SE 0.022; *P*=.68, IRR=1.009), indicating that the rate of decline did not differ significantly between the intervention and control groups. The main effect of intervention group was also not significant (b=−0.064, SE 0.145; *P*=.66, IRR=0.938), nor was the effect of fitness-club site (b=0.061, SE 0.146; *P*=.67, IRR=1.063).

**Figure 5.**
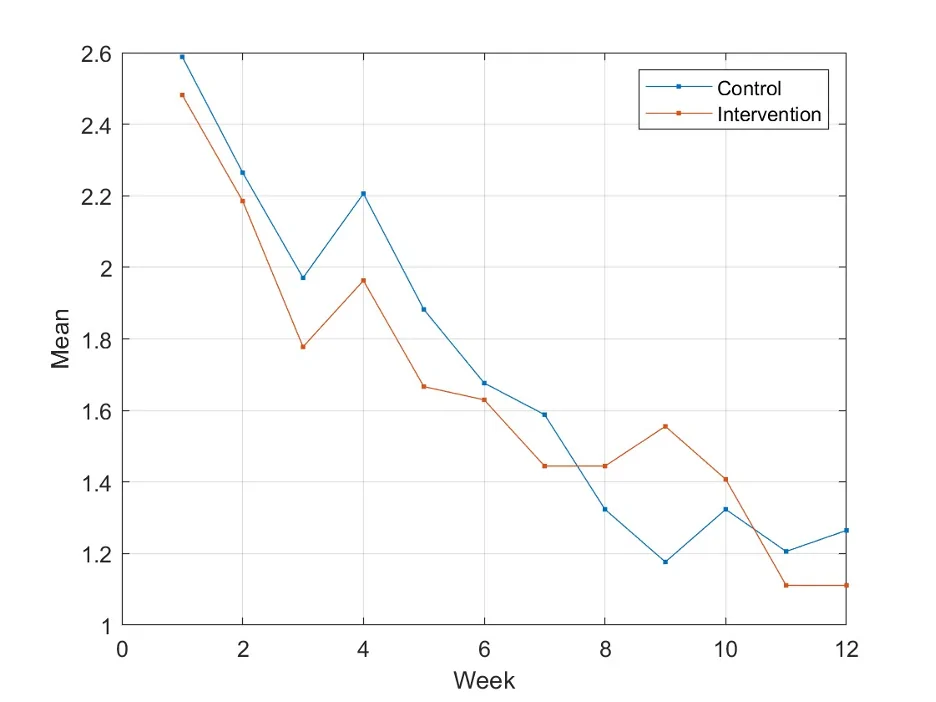
Weekly electronically recorded gym visits across the 12-week follow-up period in the intervention and control groups.

### Exploratory Comparisons by Gym Attendance Frequency

To ascertain whether individuals who visited the gym more than twice a week (n=19) on average presented a stronger average exercise habit strength than those who visited less frequently (n=42), we conducted a single-tailed *t* test, which revealed that the exercise habit strength scores for the 2 groups were significantly different, with those who exercised twice or more a week having higher scores (mean_1_ 33.89, SD 6.49) than those who exercised less (mean_2_ 28.70, SD 9.05). The assumption of equal variances was tested and found to be violated (Levene test, *P*=.01), so a single-tailed *t* test for the mean difference between samples with unequal variances was conducted (*t*_47.47_=−2.544; *P*=.02; mean difference=5.19, 95% CI 1.08-9.30). The analysis revealed a moderate effect size (*d*=0.659). When comparing the exercise self-efficacy scores at the beginning of the study (SEE at T1) between participants who exercised more than twice a week on average and those who exercised less frequently, a single-tailed *t* test revealed the following results: *t*_59_=−3.650; *P*=.001; mean difference 14.13, 95% CI 6.38-21.88 with those who exercised 2 or more times a week having higher scores (mean 71.37, SD 12.50) than those who exercised less (mean 57.24, SD 14.61). The assumption of equal variances was tested and found to be satisfied (Levene test, *P*=*.*30). The analysis revealed a large effect size (*d*=1.039); this large effect size indicates a substantial difference between the groups.

### Change in Exercise Self-Efficacy

Additionally, a paired-samples *t* test was conducted to compare the SEE scale T1 and T4 scores within each group of participants. The results indicate that the mean score at the first measurement time T1 (mean 61.64, SD 15.37) was not significantly different from the mean score at the fourth measurement time T4 (mean 58.15, SD 17.21; mean difference 3.49, 95 % CI −0.35 to 7.33) There was a moderate positive correlation between the scores at the 2 time points (*r*=0.582; *P*<.001). The analysis yielded a small effect size (*d*=0.214), suggesting a modest difference between the groups.

### Exploratory Correlations

A Pearson correlation analysis was conducted to examine the relationships among weekly gym visits, exercise habit strength, exercise self-efficacy, and exercise adherence. The results indicate significant positive correlations between all pairs of variables (Table 3). Specifically, weekly gym visits are significantly correlated with exercise habit strength (*r*=0.444; *P*<.001), exercise self-efficacy (*r*=0.498; *P*<.001), and exercise adherence (*r*=0.507; *P*<.001). The correlations among the exercise habit strength, exercise self-efficacy, and exercise adherence were also significant (*r*=0.470-0.670; all *P*<.01).

**Table 3.** Bivariate correlations among weekly gym visits, exercise habit strength, exercise self-efficacy, and exercise adherence among new adult fitness-club members included in the complete-case analysis.

Variable	Weekly gym visits	Exercise habit strength	Exercise self-efficacy	Exercise adherence
Weekly gym visits	1	0.444^a^	0.498^a^	0.507^a^
Exercise habit strength	0.444^a^	1	0.470^a^	0.509^a^
Exercise self-efficacy	0.498^a^	0.470^a^	1	0.670^a^
Exercise adherence	0.507^a^	0.509^a^	0.670^a^	1

a *P*<.01.

### Exploratory Regression Analysis

Multiple linear regression was used to predict the average weekly gym visits figure on the basis of exercise habit strength, exercise self-efficacy, exercise adherence, age, sex, education, employment, scope of employment, and marital status.

The mean weekly gym visits was 1.63 (SD 1.03). The mean scores for exercise habit strength, exercise self-efficacy, and exercise adherence were 30.32 (SD 8.63), 60.54 (SD 13.92), and 43.30 (SD 7.04), respectively.

A stepwise regression was conducted to predict weekly gym visits on the basis of several predictors, including exercise habit strength, exercise self-efficacy, exercise adherence, age, sex, education, employment, scope of employment, and marital status. The final model retained exercise adherence as the only variable retained in the exploratory stepwise model (*F*_1, 59_=20.378; *P*<.001), explaining 25.7% of the variance in weekly gym visits (*R*²=.257), and an effect size of *F*=0.35 indicated a large effect size. The regression equation was as follows:

Weekly gym visits=−1.583+0.074×EA

The exercise adherence coefficient was significant (*B*=0.074, *t*_59_=4.514, 95% CI 0.041-0.107; *P*<.001), indicating that for each unit increase in the exercise adherence score, the weekly gym visits increased by 0.074.

Overall, exercise adherence was found to be a significant exploratory variable of weekly gym visits, accounting for 25.7% of the variance. Other variables, including exercise habit strength, exercise self-efficacy, age, sex, education, employment status, scope of employment, and marital status, were not retained in the final exploratory model.

## Discussion

### Principal Findings

This study was designed as a formative evaluation intended to inform the optimization of digital health interventions rather than to provide definitive efficacy evidence. To the best of our knowledge, our study is the first to implement digital nudges using the WhatsApp app among new gym members over a multiweek period to examine whether these nudges positively affect weekly gym visit frequency. We also sought to examine how measures of self-reported exercise habit strength, exercise self-efficacy, and exercise adherence might influence and provide insights into individuals’ ability to maintain a consistent exercise routine.

### The Effect of Digital Nudging

We expected that digital prompts would positively impact visits to the gym and that higher scores on the self-reported measures would be significantly associated with better adherence to a gym exercise routine. However, the longitudinal analysis provided no evidence that the intervention altered the trajectory of gym attendance. Although weekly attendance declined significantly over the 12-week follow-up period, the group×week interaction was not significant, indicating that the rate of decline was similar in the intervention and control groups. This result may be attributed to the fact that the control group also received indirect nudges. Like the intervention group, the control group members were added to a WhatsApp group and had to complete questionnaires throughout the study period (as indicated in the timeline; Figure 1).

Several additional factors may help explain the absence of a measurable intervention effect. The 3-week intervention may have been too brief to influence gym attendance, which is shaped by multiple motivational, behavioral, and social factors. In addition, the messages were delivered in a standardized group format and were not tailored to participants’ attendance patterns, exercise goals, or perceived barriers. The study also did not assess message exposure or engagement, making it difficult to determine whether the null effect reflected limited exposure to the nudges or limited behavioral influence after exposure. These explanations were not examined directly and should therefore be interpreted cautiously.

Consequently, these findings do not indicate that the brief WhatsApp-based digital nudging intervention increased the frequency of gym visits among new members. This finding contrasts with previous studies, such as that of Calzolari and Nandardotto [14], who reported that nudging in the form of weekly digital messages, such as email reminders, significantly influences gym attendance. Additionally, Goffe and Sundamyr [15] reported that email messages were effective in increasing the frequency of gym visits by up to 9.45%. Based on these previous findings, the intervention was designed on the assumption that digital nudges could increase weekly gym attendance among new members. However, this hypothesis was not supported by the findings of this study.

### Nudges and Increasing Physical Activity

In addition, we also considered findings that demonstrated the effectiveness of nudges in improving self-management, particularly in adhering to physical activity [10]. Other studies have highlighted the effectiveness of using smartphones and digital messages to encourage physical activity and reduce sedentary behavior [12,13]. Following these findings, we concluded that leveraging digital technology could be a promising strategy for promoting healthier lifestyles and increasing physical activity levels among individuals.

### Exercise Habit Strength

To examine how measures of self-reported exercise habit strength, exercise self-efficacy, and exercise adherence might influence one’s ability to maintain a consistent exercise routine, we sought to determine whether individuals who exercised more than twice a week on average (n=19) had different exercise habit strength scores than those who exercised less often on average (n=42). Our results indicate that the mean exercise habit strength score for those who exercised less was 28.70 (SD 9.05), whereas for those who exercised more often, it was 33.89 (SD 6.49). These results show that the difference in exercise habit strength between the two groups is statistically significant (*d*=0.659; *P*=.015), suggesting that the exercise habit strength of individuals who exercise more than twice a week on average may enhance their adherence to regular exercise.

This finding is consistent with our previous narrative review, which suggested that a weekly training frequency of at least 2‐4 sessions per week is necessary to encourage habit formation [6]. Furthermore, Oliveira et al [19] demonstrated that a low training frequency (≤3 times per month) significantly increased dropout rates from 2 different gyms, and Clavel San Emeterio et al [1] reported that a frequency of more than 8 times per month increased the likelihood of staying at the gym. Therefore, fitness trainers may need to prescribe exercise routines more frequently, as this approach can increase overall exercise engagement without decreasing adherence [35]. Encouraging people to exercise more than 3 times a week may be an effective strategy to foster and strengthen physical activity habits, ultimately improving long-term adherence [7]. Additionally, recent meta-analyses using the Self-Report Habit Index scale examined the correlation between habit strength and health behaviors, underscoring the significant impact of habits on health-related actions [18]. Moreover, the results from a prior study conducted by our team revealed that veteran trainees who maintained long-term engagement in physical training exhibited an average weekly frequency of 3.95 (SD 1.56) exercise sessions [7].

### Effect of Exercise Self-Efficacy

When comparing exercise self-efficacy scores between participants who exercised more than twice a week and those who exercised less, the results show that those who exercised two or more times per week had higher scores (mean 71.37, SD 12.50) than those who exercised less (mean 57.24, SD 14.61). This finding indicates that the difference in scores between the 2 groups is statistically significant (*d*=1.039; *P*=.001). Additionally, we conducted a test to compare exercise self-efficacy scores at the beginning of the study (T1) with the average fourth measurement score (T4) to determine whether the initial exercise self-efficacy could predict long-term adherence among new gym members. Although there was a slight decrease in scores from T1 to T4, this difference was not statistically significant. This finding indicates that initial exercise self-efficacy may serve as a predictive factor for adherence to gym attendance to some extent.

### Exercise Self-Efficacy and Exercise Adherence

Perceived self-efficacy refers to an individual’s belief in their ability to successfully execute the actions necessary to navigate future situations [20]. This concept is supported by research that highlights the significant role of self-efficacy in motivating individuals to engage in physical activity [21]. Additionally, several studies have reported positive associations between self-efficacy beliefs regarding physical activity and higher levels of adherence [22,23]. This suggestion is consistent with findings from a previous study, which indicated that individuals who consistently engage in physical activity over an extended period (over a year) may have a greater perception of exercise self-efficacy [7]. Self-efficacy beliefs are important because the conviction that one can continue exercising despite obstacles such as fatigue or a busy schedule is linked to a greater likelihood of actually engaging in physical activity. According to the social-cognitive model of physical activity, self-efficacy is the primary determinant of maintaining consistent, health-promoting levels of physical activity. Individuals with higher self-efficacy regarding exercise are not only more likely to participate in an exercise program but also more aware of the benefits they expect to gain from it compared to those with lower self-efficacy [36]. Therefore, based on the aforementioned findings, it appears beneficial to assess and evaluate the self-efficacy of new gym members at the time of their initial enrollment [7].

### Frequencies of Gym Visits and Exercise Habit Strength, Exercise Self-Efficacy, and Exercise Adherence

Additionally, we examined the associations among average weekly gym visits and exercise habit strength, exercise self-efficacy, exercise adherence, and several demographic variables. In the exploratory stepwise regression model, exercise adherence was the only variable retained, and the final model explained 25.7% of the variance in average weekly gym visits (*F*_1, 59_=20.378; *P*<.001; *R*^2^=0.257). The exercise adherence coefficient was significant (B=0.074, 95% CI 0.041‐0.107), indicating that each 1-point increase in the exercise adherence score was associated with an estimated increase of 0.074 average weekly gym visits. This finding suggests that adherence-related behaviors may be relevant targets for practical strategies such as individualized goal setting, structured exercise planning, attendance monitoring, personalized feedback, and professional or social support. However, because exercise adherence and gym attendance were assessed during the same general study period, this finding should be interpreted as an exploratory association rather than evidence of temporal precedence or causality.

### Exercise Adherence

Adherence is a key aspect of behavioral change, necessitating an understanding of the processes that drive this change. Extensive literature has clarified these processes, focusing primarily on variables linked to attitudes and intentions. Among the various attitudinal determinants of behavior, self-efficacy, intentions, perceived barriers, and perceived benefits are the most reliable predictors of engagement in exercise or physical activity [24]. Lee et al [37] argue that the low rates of physical activity in the general population stem from an evolved human tendency to avoid unnecessary physical exertion.

This innate tendency developed because it allowed our ancestors to conserve energy for essential activities such as hunting, escaping predators, and engaging in social and reproductive behaviors. The common negative emotional response to physical activity is a closely related psychological mechanism that helps humans avoid wasting energy. However, this tendency does not mean that these behaviors are unchangeable. Modern human environments have eliminated the need to work for food, turning this energy conservation trait into a liability. Therefore, we have the ability to reshape our environment to make exercise necessary or, at the very least, to give it an immediate functional purpose.

Digital nudging can be viewed as an attempt to modify the immediate exercise environment by making exercise-related information and prompts more salient. In this study, exercise adherence, exercise self-efficacy, and exercise habit strength were positively associated with gym attendance. These findings are consistent with the view that exercise behavior is related not only to external prompts but also to participants’ existing behavioral and psychological characteristics.

### Role of Fitness Trainers in Exercise Adherence

Because exercise adherence involves aligning people’s attitudes with their behavior and ensuring that their behavior reflects their attitudes [24], the role of fitness trainers is crucial for developing long-lasting exercise routines. Fitness trainers can create a positive and enjoyable training environment that offers challenging exercises and fosters self-efficacy, which ultimately encourages consistency and adherence to exercise routines [6]. Consequently, as part of the practical implications for improving adherence to physical activity, it is advisable for trainers and fitness experts to provide personalized training programs that are tailored to the unique goals and preferences of each individual rather than relying on generic, one-size-fits-all approaches [7]. Festa et al [38] further recommended that future guidelines for physical activity should be personalized to better offer tailored physical activity prescriptions.

### Strengths and Limitations of This Study

Among the strengths of this study, we chose to use an online survey based on several considerations: the shorter time required to complete it (data show that these surveys take two-thirds less time to complete than traditional surveys), the ability to collect data automatically at a lower cost, automatic and permanent savings of the database, and the flexibility for participants to answer survey questions at a convenient time and location [39]. Additional strengths of this study include the use of research instruments known to have strong reliability and internal consistency. For instance, the Self-Report Habit Index is a widely accepted and validated measure across various domains, particularly with respect to habit strength [29]. Furthermore, the EARS was specifically designed to assess adherence to physical activity, making it highly relevant to the study’s focus [31], and the SEE scale [28] was designed specifically to evaluate self-efficacy in physical exercise. Additionally, the WhatsApp application is highly popular among the research population [27]. The intervention plan was constructed on the basis of existing data that showed positive results [14,15] and was framed within the EAST framework. This framework provides a straightforward evidence-based approach to effectively facilitate behavior change, assisting practitioners and policymakers in the practical application of behavioral insights [17]. Another strength of the study is its design, which involved the collection of personal indicators and self-reports throughout the research period, offering comprehensive insights into the study population and their behaviors. A major limitation of this study was postenrollment questionnaire noncompletion and the resulting reliance on complete-case analyses. As shown in Figure 2, individuals verbally expressed initial willingness to participate and were provisionally assigned, but only 94 completed electronic informed consent and the T1 questionnaire and were formally enrolled. The other 106 individuals should therefore not be considered participants lost to follow-up. Among the 94 formally enrolled participants, 61 (64.9%) completed all 4 assessments and were included in the complete-case analytic sample, whereas 33 (35.1%) did not. Specific reasons for questionnaire noncompletion were not systematically recorded. Consequently, incomplete questionnaire follow-up cannot be interpreted as confirmed discontinuation of gym membership or exercise participation.

Gym-entry records required manual retrieval by fitness-club staff and were extracted only for the 61 participants included in the complete-case sample. Attendance data for the other 33 consented participants were therefore unavailable for the current analysis. The primary gym attendance analysis could not consequently include all formally enrolled participants, introducing a risk of selection bias if questionnaire completers differed from noncompleters in their attendance behavior or study engagement. Although incomplete follow-up has also been reported in studies with comparable designs [11,40], the reduced analytic sample limits generalizability and may have reduced statistical power. Repeated smartphone-based questionnaires and reminders may have contributed to participant burden [41]. Future studies should prospectively retrieve attendance records for all consented participants, independently of questionnaire completion, and use shorter questionnaires, fewer assessment points, individualized reminders, and predefined retention and missing data procedures.

A further limitation concerns possible measurement reactivity in the control group. Although control participants received no nudge messages, they were included in a WhatsApp group and completed repeated study questionnaires. This process may have prompted reflection on their exercise behavior and influenced their engagement, potentially reducing the observed differences between the study groups [11].

### Implications and Future Research

Future studies should examine multicomponent adherence programs that combine digital support with individualized goal setting, professional guidance, attendance monitoring, personalized feedback, social support, group-based activities, and appropriate incentives. Intervention content and delivery could also be tailored to participants’ exercise goals, attendance patterns, and perceived barriers. Larger samples and longer follow-up periods are needed to determine whether such adaptive and personalized approaches can support sustained gym attendance and exercise adherence.

### Conclusions

Maintaining regular exercise among new gym members is a complex behavioral process that is unlikely to be sustained through brief external reminders alone. Although the WhatsApp-based digital nudging intervention did not increase weekly gym visits, higher exercise adherence, exercise self-efficacy, and exercise habit strength were associated with more frequent attendance. Participants attending at least twice weekly also reported greater self-efficacy and stronger exercise habits. Although these findings should be interpreted as exploratory rather than causal, they identify potentially relevant behavioral factors for understanding and supporting continued exercise participation among new gym members.

## Abbreviations

EARS: Exercise Adherence Rating Scale
EAST: Easy, Attractive, Social, and Timely
GEE: generalized estimating equations
IRR: incidence rate ratio
SEE: self-efficacy for exercise
